# Genome-Wide Characterization and Expression Analysis of Major Intrinsic Proteins during Abiotic and Biotic Stresses in Sweet Orange (*Citrus sinensis* L. Osb.)

**DOI:** 10.1371/journal.pone.0138786

**Published:** 2015-09-23

**Authors:** Cristina de Paula Santos Martins, Andresa Muniz Pedrosa, Dongliang Du, Luana Pereira Gonçalves, Qibin Yu, Frederick G. Gmitter, Marcio Gilberto Cardoso Costa

**Affiliations:** 1 Center for Biotechnology and Genetics, Biological Sciences Department, State University of Santa Cruz, Ilhéus, Bahia, Brazil; 2 Citrus Research and Education Center, Department of Horticultural Sciences, University of Florida, Lake Alfred, Florida, United States of America; USDA-ARS-SRRC, UNITED STATES

## Abstract

The family of aquaporins (AQPs), or major intrinsic proteins (MIPs), includes integral membrane proteins that function as transmembrane channels for water and other small molecules of physiological significance. MIPs are classified into five subfamilies in higher plants, including plasma membrane (PIPs), tonoplast (TIPs), NOD26-like (NIPs), small basic (SIPs) and unclassified X (XIPs) intrinsic proteins. This study reports a genome-wide survey of *MIP* encoding genes in sweet orange (*Citrus sinensis* L. Osb.), the most widely cultivated *Citrus* spp. A total of 34 different genes encoding *C*. *sinensis* MIPs (CsMIPs) were identified and assigned into five subfamilies (CsPIPs, CsTIPs, CsNIPs, CsSIPs and CsXIPs) based on sequence analysis and also on their phylogenetic relationships with clearly classified MIPs of *Arabidopsis thaliana*. Analysis of key amino acid residues allowed the assessment of the substrate specificity of each CsMIP. Gene structure analysis revealed that the *CsMIPs* possess an exon-intron organization that is highly conserved within each subfamily. *CsMIP* loci were precisely mapped on every sweet orange chromosome, indicating a wide distribution of the gene family in the sweet orange genome. Investigation of their expression patterns in different tissues and upon drought and salt stress treatments, as well as with ‘*Candidatus* Liberibacter asiaticus’ infection, revealed a tissue-specific and coordinated regulation of the different *CsMIP* isoforms, consistent with the organization of the stress-responsive *cis*-acting regulatory elements observed in their promoter regions. A special role in regulating the flow of water and nutrients is proposed for *CsTIPs* and *CsXIPs* during drought stress, and for most *CsMIPs* during salt stress and the development of HLB disease. These results provide a valuable reference for further exploration of the *CsMIPs* functions and applications to the genetic improvement of both abiotic and biotic stress tolerance in citrus.

## Introduction

Aquaporins (AQPs) are integral membrane proteins that assist the rapid movement of water as well as other low molecular weight molecules across cellular membranes [1–3]. AQPs belong to the ancient family of major intrinsic proteins (MIPs) found in microorganisms, plants and animals. While a small number of different AQPs have been identified (2 in *E*. *coli*, 9 in *S*. *cerevisiae*, 11 in *C*. *elegans* and 13 in mammals [4]), a surprisingly large number of MIP homologues have been found in plants; for example, 35 AQPs were found in *Arabidopsis* [5], 36 in *Zea mays* [6], 33 in *Oryza sativa* [7], 28 in *Vitis vinifera* [8], 55 in *Populus trichocarpa* [9], 71 in *Gossypium hirsutum* [10], 47 in *Solanum lycopersicum* [11] and 66 in *Glycine max* [12]. These observations highlight a major role for plant MIPs as key regulators of the intricate flows of water and solutes required for growth and adaptive responses to the ever-changing environment.

Plant MIPs were originally categorized into four subfamilies on the basis of sequence homologies and subcellular localization: plasma membrane (PIP), tonoplast (TIP), nodulin-like (NIP) and small basic (SIP) intrinsic proteins [2,13]. More recently, studies in the moss *Physcomitrella patens* revealed the presence of novel AQP isoforms in addition to the four conserved plant AQP subfamilies: a homologue of the *Escherichia coli* intrinsic protein GlpF (GIPs), intrinsic hybrid proteins (HIPs) and unclassified X intrinsic proteins (XIPs) [9,13–17]. XIP homologues have also been identified in some higher plants, such as *Solanum lycopersicum*, *Populus trichocarpa* and *Glycine max* [9,11,12,15]. These findings suggest that the family of plant MIPs is larger and much more complex than previously anticipated and, hence, may play critical roles in a wide range of biological processes that go far beyond the current knowledge.

AQP-mediated water transport in plants has been implicated to play a central regulatory step in diverse biological processes, including cell elongation, seed germination and osmoregulation [18]. In addition, AQPs facilitate the transport of small uncharged molecules of physiological significance like glycerol, urea, boric acid, silicic acid, hydrogen peroxide (H_2_O_2_), ammonia (NH_3_) and carbon dioxide (CO_2_) through the plant cell membranes [1,2] and also regulate phloem sap loading and unloading, stomatal and leaf movement, and cytoplasmic homeostasis [1,2,13,19]. Therefore, it is not surprising that their expression and biological activities have been shown to be developmentally and differentially regulated in a cell-specific manner, via phytohormones such as abscisic acid (ABA), gibberellins and possibly brassinosteroids, and by environmental signals such as light, water stress, nematode infection, low temperature, and salinity [4]. However, a general expression pattern among the distinct MIP isoforms cannot be distinguished, as they are either up- or downregulated depending on the stimulus and/or the cell-type studied [4,19,20]. This difference in transcriptional regulation suggests that each MIP isoform may play a distinct role in plant growth, development and stress response [4].

As a major horticultural crop, the cultivated *Citrus* spp. face constant biotic and abiotic constraints in the main regions of production, including drought, salinity, extreme temperatures and serious diseases like Huanglongbing (HLB, or citrus greening), which are predicted to increase in intensity, frequency, and geographic extent as a consequence of global climate change. Despite the highlighted importance of AQPs, there are only a few studies to date on citrus MIPs and their predicted role in the transport of water and solutes required for plant growth, development and adaptive responses to the environment. The expression of two *MIP* genes, *PIP1* and *PIP2*, has been investigated in roots of *Poncirus trifoliata* (L.) Raf., Cleopatra mandarin (*C*. *reshni* Hort exTan.) and one of their hybrids, subjected to moderate water deficit [21], and in roots of *P*. *trifoliata*, Cleopatra mandarin and Carrizo citrange (*C*. *sinensis* [L.] Osb.×*P*. *trifoliata* [L.] Raf.), subjected to salt treatment [22]. *PIP1* and *PIP2* gene expression differences were correlated with alterations in root hydraulic conductance (Kr) and chloride (Cl^-^) exclusion from leaves and, hence, tolerance to water and salt stresses, respectively. With the recent completion and publication of the genome sequences of sweet orange [23–25], it is now possible to identify and characterize the complete repertoire of MIPs in citrus, as well as to carry out comparative genome analysis in order to understand their evolutionary history. Therefore, the objective of the present study was to identify sweet orange *MIP* genes through a genome-wide analysis and to characterize their sequences, evolutionary relationships, putative functions and expression patterns in various tissues and in response to abiotic and biotic stresses. This is the first comprehensive study of the *MIP* gene family in sweet orange, providing valuable information for further exploration of the functions of this important gene family in citrus.

## Materials and Methods

### Plant materials and stress treatments

Two-year-old sweet orange plants grafted on Rangpur lime (*C*. *limonia* Osbeck), a rootstock highly resistant to drought, were used in the drought stress experiment. Plants were first pruned and acclimatized to greenhouse conditions (25±4°C, 16 h of light and relative humidity oscillating between 80 and 95%) during 90 days to obtain adequate root development and uniform leaf flushes. During acclimatization, plants were grown in plastic pots of 45-L, containing a mixture of soil and sand (ratio 3:1) and micronutrient mix FTE (50g per pot), irrigated with tap water twice a week, and fertilized weekly with 1 liter of the following nutrient solution: 1.0g l^-1^ Ca(NO_3_)_2_, 0.4g l^-1^ KNO_3_, 0.6g l^-1^ MgSO_4_ and 0.4g l^-1^ NH_4_H_2_P0_4_ (MAP). Thereafter, the pots were closed with aluminum foil to prevent water loss by evaporation, and a set of 10 plants was randomized over the experimental area and subjected to the following treatments: (i) 5 plants in control, in which plants were maintained at leaf predawn water potential values of -0.2 to -0.4 MPa by daily irrigation and (ii) 5 plants in drought, in which the plants were exposed to a progressive soil water deficit until their leaves reach predawn water potential values of -1.5 MPa. The leaf predawn water potential was recorded on the third fully expanded mature leaf from the apex of each plant, between 2 AM and 4 AM, using a Scholander-type pressure pump (m670, Pms Instrument Co., Albany, USA).

For salt treatment, sweet orange seeds were germinated in vitro as described by de Oliveira et al. [26]. Twenty-day-old seedlings of nucellar origin were selected based on their uniformity, and transferred to MS medium alone (control) or containing 150 mM NaCl (Merck, Darmstadt, Germany). Each treatment consisted of 15 nucellar plants (biological replicates). Leaves and roots were harvested 20 days after the treatments and immediately frozen in liquid nitrogen and stored at -80°C.

Plants were infected with ‘*Candidatus* Liberibacter asiaticus’ as described in Fan et al. [27]. Briefly, two-year-old seedlings of rough lemon (*C*. *jambhiri Lush*.) and sweet orange (*C*. *sinensis L*. *Osbeck*) were graft-inoculated with bud wood from ‘*Ca*. L. *asiaticus*’ infected ‘Carrizo’ citrange trees kept under greenhouse conditions. For controls, the plants were grafted with bud wood from healthy Carrizo trees. All these plants were kept in a United States Department of Agriculture Animal and Plant Health Inspection Service and Center for Disease Control-approved and secured greenhouse at the University of Florida, Citrus Research and Education Center, Lake Alfred. Three biological replicates were produced for each citrus species in each treatment. Quantitative real-time PCR was performed to confirm the presence of ‘*Ca*. L. *asiaticus*’ in the inoculum source and inoculated plants as described in Li et al. [28]. Four fully expanded leaves were sampled separately from ‘*Ca*. L. *asiaticus*’ inoculated plants and mock-inoculated plants (used as controls) of each citrus species at 0, 7, 17, and 34 weeks after inoculation (WAI). Leaves were immediately frozen in liquid nitrogen and stored at -80°C until use. Three biological replicates were produced for each condition. In total, 12 plants with 48 leaf samples were collected (2 species x 2 treatments x 3 replicates x 4 time points).

### Identification and classification of CsMIPs

The Hidden Markov Model profile of the PFAM (http://pfam.sanger.ac.uk/) [29] motif PF00230 (major intrinsic protein) was used as a keyword to search the sweet orange genome sequence database (http://www.phytozome.org/citrus/) [25]. The KEGG Orthology (KO) terms K09872 (aquaporin PIP), K09873 (aquaporin TIP), K09874 (aquaporin NIP) and K09875 (aquaporin SIP) were also used as keywords to search the sweet orange genome sequence at Phytozome. To avoid the deficiencies of the automatic annotation, the 35 *Arabidopsis thaliana* MIP protein sequences were retrieved from TAIR (http://www.arabidopsis.org/), according to previous reports [5,30], and also used to align with the sweet orange genome sequence assembly available at Phytozome using the TBLASTN tool. After merging the results from all these strategies, unique entries (with unique locus ID) were identified to remove the redundancy. The resulting sequences were manually inspected for the presence of characteristic and functionally important MIP amino acids and motifs.

The sweet orange MIPs were classified in different isoforms based on sequence analysis of the multiple alignments and on their phylogenetic relationship with those clearly classified MIPs of *Arabidopsis thaliana*, *Ricinus communis* and *Nicotiana benthamiana*, downloaded from the TAIR and NCBI databases. Multiple sequence alignments of the deduced amino acid sequences of CsMIPs and those of *A*. *thaliana*, *R*. *communis* and *N*. *benthamiana* were performed using the default parameters of ClustalW [31]. The dendrogram was generated by the MEGA 6 program [32] using the Neighbor-Joining (NJ) method [33] and bootstrap analysis (1,000 replications).

### Analysis of CsMIP protein properties and conserved amino acid residues

Information about coding sequence (CDS), full-length sequence and predicted amino acid sequence was obtained for each sweet orange *MIP* gene from the Phytozome database. The GRAVY (grand average of hydropathy), molecular weight and isoelectric point (pI) of the deduced amino acid sequences were predicted by the PROTPARAM tool available on the Expert Protein Analysis System (ExPASy) proteomics server (www.expasy.ch/tools/protparam.html). The subcellular localization of MIP proteins was predicted using the WoLF PSORT tool available at http://www.genscript.com/psort/wolf_psort.html. Careful visual inspection of amino acid sequence alignments were performed to identify the characteristic MIP amino acids and motifs and the residues in seven key positions that have been reported to be specific for each functional subgroup [12,30,34,35].

### Analysis of promoter sequences and chromosomal locations of *CsMIPs*

One kb upstream region from the translation start site was extracted from all the sweet orange *MIP* genes and subsequently analyzed in the PLACE database (http://www.dna.affrc.go.jp/PLACE/signalscan.html) to identify the presence of the stress-responsive *cis*-acting regulatory elements ABRE (ABA-responsive element; ACGTG), DRE/CRT (dehydration responsive element/C-repeat; G/ACCGAC), MYBS (MYB binding site; TAACTG) and LTRE (low-temperature-responsive element; CCGAC) in their promoters. The physical locations of *CsMIPs* were determined by confirming the starting position of all genes on each chromosome, using BLASTN searching against the local database of the *Citrus sinensis* Annotation Project (CAP; http://citrus.hzau.edu.cn/orange/). MapChart software was used to plot the gene loci on the sweet orange chromosomes [36].

### Expression analysis of *CsMIPs*

Total RNA isolation, cDNA synthesis and quantitative real-time RT-PCR (qPCR) analysis were performed as described previously [26]. qPCR primers were designed appropriately to avoid the conserved regions. Primer sequences are shown in detail in S1 Table. Glyceraldehyde-3-phosphate dehydrogenase C2 (*GAPC2*) was used as an internal reference gene to normalize expression among the different samples [37]. Data were obtained from a pool of three biological replicates that were individually validated.

RNA-seq data were downloaded from CAP [24] and used to analyze the expression patterns of *CsMIPs* in different tissues, namely callus (C), flower (Fl), leaf (L), fruit (Fr), and mixed tissues from fruit at three developmental stages (Mix.1, Mix.2, and Mix.3). The heatmap was generated using R 3.1.0 software.

## Results and Discussion

### MIP encoding genes in the sweet orange genome

Searches in the sweet orange genome sequence database at Phytozome using annotation information, as well as the 35 protein sequences of the complete set of *A*. *thaliana* MIPs (AtMIPs) as query sequences, resulted in the identification of 34 different genes encoding *C*. *sinensis* MIPs (CsMIPs) (Table 1). The retrieved sequences were manually inspected for the presence of characteristic and functionally important MIP domains and motifs, such as the highly conserved NPA (Asn-Pro-Ala) motifs, and considered to be correct. The number of *MIP* genes described in this study is similar to that found in the genomes of *Arabidopsis* [5], maize [6], rice [7] and grape [8], but significantly lower than that identified in the genomes of poplar [9], cotton [10], tomato [11] and soybean [12]. The absence of recent whole-genome duplication (WGD) events in the sweet orange genome, as described by Xu et al. [23], could account for the relatively small size of the *MIP* family in the citrus genome.

**Table 1:** Genes and encoded MIP proteins in sweet orange.

Gene name	Locus	Chromosome location	Group	Kegg Orth ID	Polypeptide length (MW)	pI	GRAVY	Predicted subcellular localization
CsPIP1;1	orange1.1g018895m	chr7: 31,253,722…31,256,103	PIP	K09872	349 (37.56kDa)	9.39	0.397	plasma membrane
CsPIP1;2	orange1.1g023021m	chr7: 31,247,369…31,248,902			288 (30.67kDa)	7.71	0.414	plasma membrane
CsPIP1;3	orange1.1g023107m	chr5: 1,804,896…1,807,677			287 (30.70kDa)	8.97	0.344	plasma membrane
CsPIP1;4	orange1.1g023069m	chr6: 9,907,825…9,909,541			287 (30.82kDa)	8.96	0.343	plasma membrane
CsPIP2;1	orange1.1g023108m	chr6: 12,833,884…12,835,042			287 (30.67kDa)	8.74	0.376	plasma membrane
CsPIP2;2	orange1.1g022966m	chr8: 19,657,287…19,659,497			289 (31.05kDa)	7.62	0.392	plasma membrane
CsPIP2;3	orange1.1g019681m	chr7: 26,202,220…26,205,546			337 (36.34KDa)	9.77	0.403	plasma membrane
CsPIP2;4	orange1.1g023370m	chr8: 981,841…983,571			283 (30.14kDa)	8.99	0.431	plasma membrane
CsTIP1;1	orange1.1g025548m	chrUn: 46,663,407…46,665,011	TIP	K09873	251 (26.06kDa)	6.12	0.675	vacuole
CsTIP1;2	orange1.1g025600m	chr8: 20,659,157…20,660,437			250 (25.65kDa)	5.32	0.841	cytosol
CsTIP1;3	orange1.1g037978m	chr8: 20,659,157…20,660,437			124 (12.92kDa)	4.37	0.734	cytosol
CsTIP1;4	orange1.1g025464m	chr7: 29,135,182…29,136,531			252 (26.01kDa)	5,69	0,786	vacuole
CsTIP2;1	orange1.1g025817m	chr1: 18,627,617…18,629,472			247 (25.15kDa)	5.59	0.894	vacuole
CsTIP2;2	orange1.1g025865m	chr1: 18,627,617…18,629,472			247 (25.10kDa)	5.59	0.902	vacuole
CsTIP2;3	orange1.1g038895m	chr5: 5,749,487…5,750,939			206 (20.55kDa)	4.72	0.979	vacuole
CsTIP3;1	orange1.1g025197m	chr5: 16,938,542…16,940,192			256 (26.99kDa)	7.07	0.626	cytosol
CsTIP4;1	orange1.1g025864m	chr4: 19,032,254…19,033,990			247 (16.27kDa)	6.27	0.825	vacuole
CsTIP5;1	orange1.1g046726m	chr9: 14,144,215…14,145,199			161 (16.86kDa)	9.00	0.770	cytosol
CsTIP6;1	orange1.1g042738m	chr9: 14,144,215…14,145,199			107 (11.11kDa)	4.54	0.636	secreted
CsNIP1;1	orange1.1g023184m	chr2: 2,151,220…2,153,387	NIP	K09874	286 (30.45kDa)	8.64	0.434	vacuole
CsNIP2;1	orange1.1g036721m	chr6: 18,134,848…18,136,228			223 (23.70kDa)	9.69	0.889	cytosol
CsNIP2;2	orange1.1g040981m	chr6: 18,134,848…18,136,228			211 (22.32kDa)	9.39	0.967	cytosol
CsNIP2;3	orange1.1g040755m	chr2: 13,464,261…13,465,771			275 (29.39kDa)	8.88	0.628	plasma membrane
CsNIP3;1	orange1.1g023102m	chr6: 20,482,599…20,486,038			287 (30.30kDa)	8.40	0.387	plasma membrane
CsNIP4;1	orange1.1g046511m	chr3: 23,770,831…23,774,009			282 (29.28kDa)	8.84	0.372	vacuole
CsNIP5;1	orange1.1g035030m	chr1: 13,680,675…13,682,992			75 (7.75kDa)	8.98	0.291	chloroplast
CsNIP5;2	orange1.1g027840m	chr1: 13,678,241…13,681,090			218 (22.51kDa)	7.75	0.462	plasma membrane
CsNIP6;1	orange1.1g039196m	chr9: 3,798,017…3,800,780			288 (30.20kDa)	7.53	0.718	plasma membrane
CsSIP1;1	orange1.1g026039m	chr5: 28,968,880…28,972,401	SIP	K09875	244 (25.92kDa)	9.35	0.727	plasma membrane
CsSIP1;2	orange1.1g026082m	chr3: 1,234,876…1,236,556			244 (26.17kDa)	9.83	0.749	plasma membrane
CsSIP2;1	orange1.1g026600m	chr6: 17,078,102…17,081,323			236 (25.57kDa)	9.70	0.600	chloroplast
CsXIP1;1	orange1.1g036381m	chr8: 7,139,938…7,141,114	XIP	-	235 (25.09kDa)	8.70	0.821	plasma membrane
CsXIP1;2	orange1.1g040654m	chr8: 7,131,064…7,132,799			302 (32.68kDa)	8.74	0.573	plasma membrane
CsXIP2;1	orange1.1g045670m	chr8: 7,128,184…7,129,448			319 (34.58kDa)	8.32	0.681	plasma membrane

The CsMIPs were classified in five different subfamilies, PIPs, TIPs, NIPs, SIPs and XIPs, based on analysis of the amino acid residues located in seven key positions (P1 to P7) that were previously proposed [12,34,35] to discriminate the different subfamilies (S2 Table), as well as on their phylogenetic relationships with the well classified MIPs of *A*. *thaliana* and XIPs of *R*. *communis* and *N*. *benthamiana* (S1 Fig). Our analysis revealed the presence of 8 PIPs, 11 TIPs, 9 NIPs, 3 SIPs and 3 XIPs in the sweet orange genome (Table 1). The CsMIPs were named according to the nomenclature proposed in classification of the MIPs of *A*. *thaliana*. This nomenclature was based on phylogenetic analyses and where the names in a systematic way reflect distinct clades that are evolutionarily stable [5]. PIPs, TIPs, NIPs and SIPs from sweet orange grouped with their respective *Arabidopsis* counterparts, indicating the large extent of conservation between the sweet orange and *Arabidopsis MIP* gene families (S1 Fig). The only exception was CsTIP6;1, which was found to encode a N- and C-terminally truncated TIP protein compared to the rest of the subfamily.

To examine whether the number of *MIP* genes found in the diploid sweet orange is comparable to that of the dihaploid sweet orange and haploid Clementine mandarin, we also performed homology searches against the dihaploid sweet orange draft genome available at the *Citrus sinensis* Annotation Project (CAP) and the reference haploid Clementine mandarin (*C*. *clementina*) genome available at Phytozome (S3 Table). Although the total number of *MIP* genes was roughly similar among the different citrus genomes, significant differences were observed in the number of members within the subfamilies (Table 2). BLAST similarity analysis revealed that the dihaploid sweet orange and haploid Clementine contained two additional *PIP* isoforms closely related to the *CsPIP2;1* (S3 Table). Clementine also contained one additional *PIP* isoform closely related to the *CsPIP1;2* and one *PIP*, *TIP* and *NIP* isoform without homology to any *MIP* sequence from the diploid sweet orange (S3 Table). *CsTIP1;2* and *CsTIP1;3*, *CsTIP2;1* and *CsTIP2;2*, *CsTIP5;1* and *CsTIP6;1*, and *CsNIP2;1* and *CsNIP2;2* exhibited significant hits to the same *MIP* isoforms of the dihaploid sweet orange and haploid Clementine (S3 Table). These observed variations in the size of the *MIP* subfamilies may be a consequence of the different sequencing depth and assembly quality between the diploid and dihaploid sweet orange genomes [23,25] and the evolutionary origin of Clementine, which is a hybrid of Willowleaf mandarin and sweet-orange [25] and, thereby, it contains more *C*. *reticulata* haplotype regions than found in sweet orange.

**Table 2:** Comparison of the number of the different MIP family members in diploid (Phytozome) and dihaploid (CAP) sweet oranges (*C*. *sinensis*) and haploid Clementine (*C*. *clementina*).

Subfamily	Diploid *C*. *sinensis*	Dihaploid *C*. *sinensis*	*C*. *clementina*
PIP	8	10	11
TIP	11	8	9
NIP	9	8	8
SIP	3	3	3
XIP	3	3	2
**Total**	**34**	**32**	**33**

### CsMIP protein properties and conserved amino acid residues

The CsMIPs encode proteins ranging from 75 (7.7 kDa) to 349 (37.6 kDa) amino acids in length, and pI values ranging from 4.37 to 9.77 (Table 1). The average protein length of PIPs, TIPs, NIPs, SIPs and XIPs were 300.8 (32.2 kDa), 213.4 (21.1 kDa), 238.3 (25.1 kDa), 241.3 (25.9 kDa) and 285.3 (30.8 kDa) amino acids, respectively. The average pI of PIPs, TIPs, NIPs, SIPs and XIPs were 8.77, 5.86, 8.68, 9.63, and 8.59, respectively. These data reveal that CsTIPs are not only smaller, but most of them are also much more acidic than the other CsMIPs, as reported for *Arabidopsis* MIPs [38]. The cause of these large differences in TIPs has been attributed to the smaller amount of basic residues found at the carboxyl termini of TIPs compared with the other MIPs [5]. All the CsMIPs had a positive GRAVY score (Table 1), suggesting that they are hydrophobic proteins, which is a necessary characteristic for AQPs [1]. Analysis of the predicted subcellular localization showed that all CsPIPs and CsXIPs were localized to plasma membrane (Table 1). The predicted localization of CsTIPs, CsNIPs and CsSIPs was more diverse, including vacuole (CsTIP1;1, CsTIP1;4, CsTIP2;1, CsTIP2;2, CsTIP2;3, CsTIP4;1, CsNIP1;1 and CsNIP4;1), cytosol (CsTIP1;2, CsTIP1;3, CsTIP3;1, CsTIP5;1, CsNIP2;1 and CsNIP2;2), plasma membrane (CsNIP2;3, CsNIP3;1, CsNIP5;2, CsNIP6;1, CsSIP1;1 and CsSIP1;2), chloroplast (CsNIP5;1 and CsSIP2;1) and secreted (CsTIP6;1) (Table 1). These results seem to be in agreement with the experimentally determined localizations of MIPs reported in the literature [13,39].

MIP folding is characterized by six transmembrane α-helices (H1 to H6) that are connected by five loops (loops A-E), forming an aqueous transmembrane pore that constitutes the functional core of MIPs [35]. Loops B (LB) and E (LE) contain two highly conserved NPA (Asn-Pro-Ala) motifs that are considered to be critical for the substrate selectivity of MIPs [40,41]. Another set of four conserved residues forms the aromatic/Arginine selectivity filter (ar/R filter), which has been proposed to act as a size exclusion barrier for substrate molecules [42]. The first two residues are located in H2 and H5, while the latter two are found in LE (LE1 and LE2). Finally, seven key amino acid residues (named positions P1 to P7) have been proposed to discriminate the five subfamilies [12,34]. P1 is located in the terminal part of H3, while P2 and P3 are located in LE, just behind the second NPA motif (2^nd^ and 6^th^ residues after 2^nd^ NPA, respectively). P4 and P5 correspond to two consecutive amino acids located in H6, while P6 and P7 also correspond to two consecutive amino acids located in H3. The multiple sequence alignments were carefully analyzed and all these conserved motifs and amino acid residues were identified in most CsMIPs, indicating that they are functional channels for water and other solutes (S2 Table). All the CsPIPs showed the dual typical NPA motifs and an ar/R filter configuration characteristic for a water-transporting MIP (F,H,T,R). Additional presence of the S-A-F-W residues at P2-P5 positions, as observed in all CsPIPs, except for CsPIP1;1, has been interpreted as a signature of CO_2_ transporter PIPs [35]. All the CsTIPs also had the two canonical NPA motifs, except CsTIP1;3, CsTIP5;1 and CsTIP6;1, which were found to encode truncated proteins lacking either the first (CsTIP1;3) or the second NPA motif (CsTIP5;1 and CsTIP6;1), as well as other conserved amino acid residues of the ar/R filter region (S2 Table). TIPs containing the H-I-A-V or H-I-G-R residues in the ar/R filter and T-A-A-Y-W or T-S-A-Y-W residues at P1-P5 positions, like CsTIP1s, CsTIP2s and CsTIP3, have been shown to transport urea and H_2_O_2_ [35]. The CsNIP1;1, CsNIP2;1, CsNIP2;2 and CsNIP2;3 showed an ar/R filter configuration identical to that of soybean Nodulin 26, indicating that they are also able to facilitate water and solute transport capability [30]. The residue at the H5 position of the ar/R filter of AtNIP5;1 was shown to play a key role in the membrane permeability to water, silicic acid (Si) and boric acid (B) [42]. AtNIP5;1 with AIGR residues for the ar/R filter was shown to transport water, B and arsenite (As), but not Si [42]. CsNIP3;1, with GSGR residues for ar/R filter, can be expected to transport water, Si and B, while CsNIP4;1 (AIGR residues) may transport water, B and As. The CsSIPs showed a less conserved first NPA motif, while the second NPA motif was perfectly conserved in all members (S2 Table). AtSIP1 isoforms, but not AtSIP2;1, were shown to be functional water channels [43], suggesting that the latter may be involved in the transport of solutes. However, SIP transport function and structural organization still await biochemical characterization. The CsXIPs also showed a modified first NPA motif, NPL (CsXIP1s) or SPV (CsXIP2), and a conserved second NPA motif (S2 Table). The four positions in the ar/R filter region contained amino acid residues that were strictly conserved among the CsXIPs. CsXIP1;1 was observed to contain an internal deletion of 13 amino acid residues in the H2 region that abolished the conserved amino acid V at position H2 of the ar/R selective filter. Since the first three amino acid of the ar/R filter have rather hydrophobic residues (VVAR or VVVR), the CsXIPs might be involved in the transport of molecules other than water [35]. In fact, a recent study has indicated that the Solanaceae XIPs are plasma membrane aquaporins involved in the transport of many uncharged substrates, such as urea, H_2_O_2_ and B [44].

### Genomic organization of *CsMIPs*

The exon-intron structure of all 34 *CsMIP* genes was analyzed using the sweet orange gene models annotated in Phytozome. With a few exceptions, the number and size of the exons, but not of the introns, were observed to be conserved within each CsMIP subfamily (S2 Fig). All the *CsPIPs* presented three introns and four exons, as reported for all *Arabidopsis* [5], poplar [9], tomato [11] and soybean PIPs [12]. The majority of *CsTIPs* contained two introns and three exons, with exception of *CsTIP1;1*, *CsTIP2;3* and *CsTIP6;1*, which showed one intron and two exons, and the truncated *CsTIP1;3* (one exon) gene. Such a more varied pattern of exon-intron structure has been also observed in *Arabidopsis* [5], poplar [9], tomato [11] and soybean *TIPs* [12]. Most *CsNIPs* contained four introns and five exons, like all *Arabidopsis* [5] and most poplar [9], tomato [11] and soybean NIPs [12]. The exceptions were *CsNIP2;2* (three introns and four exons), *CsNIP5;2* (two introns and three exons) and the truncated *CsNIP5;1* (one intron and two exons) genes. The *CsSIPs* featured two introns and three exons, except *CsSIP1;2* (one exon and one intron in the 3’-UTR region). Similar patterns of exon-intron structure were also reported for tomato SIPs [11], while all the *Arabidopsis* [5] and poplar SIPs [9] had two introns and three exons. The gene structure of *CsXIPs* varied among all members, which contained two introns and three exons (*CsXIP1;2*), either one intron and two exons (*CsXIP2;1*) or only one exon (*CsXIP1;1*). The pattern of two intron and three exons has been reported for most poplar [9] and tomato XIPs [11], while all soybean XIPs contained a single intron and two exons [12].

The positions of all 34 *CsMIPs* were mapped on the sweet orange chromosomes by homology searches against the full-length sweet orange genome assembly available at the CAP database (Table 1 and S3 Table). Except for *CsTIP1;1*, which was not exactly located on any chromosome because of an incomplete physical map for sweet orange, all the *CsMIP* loci were precisely mapped on every sweet orange chromosome, indicating a wide distribution of the gene family in the sweet orange genome (Table 1 and S3 Fig). The closely related *CsMIP* isoforms *CsTIP1;2* and *CsTIP1;3*, *CsTIP2;1* and *CsTIP2;2*, *CsTIP5;1* and *CsTIP6;1*, and *CsNIP2;1* and *CsNIP2;2* were respectively mapped on identical chromosome positions since they showed significant hits to the same genes in the CAP database (S3 Table). Seven *CsMIPs* were found to be tandem duplicated genes according to the criteria of Hanada et al. [45], which defined tandem duplicates as genes in any gene pair, T_1_ and T_2_, that (1) belong to the same gene family, (2) are located within 100 kb each other, and (3) are separated by 10 or fewer nonhomologous spacer genes. These were *CsNIP5;1* and *CsNIP5;2* on chromosome 1, *CsPIP1;1* and *CsPIP1;2* on chromosome 7, and *CsXIP1;1*, *CsXIP1;2* and *CsXIP2;1* on chromosome 8 (Table 1 and S3 Fig). These results suggest that all these *CsMIPs* may have evolved from tandem duplication events, as also recently proposed for the tomato *XIPs* [11].

Analysis of previously mapped traits revealed that the 282-kb region surrounding the Citrus Tristeza Virus resistance (*Ctv*) locus is physically linked (~40-kb) to the *CsNIP5;1* and *CsNIP5;2* genes, on the chromosome 1 [46]. This region was also reported to contain *Tyr1*, the major locus controlling citrus nematode (*Tylenchulus semipenetrans*) resistance [47,48].

### Expression patterns of *CsMIP* genes in different tissues

To investigate the expression patterns of *CsMIPs* in different tissues, RNA-seq data were downloaded from CAP [24]. The heatmap generated showed a differential transcript abundance of the 34 *CsMIPs* in four major tissues, namely callus, flower, leaf, fruit, and mixed fruit tissues at three developmental stages (S4 Fig). Some of the *CsMIPs* (*CsPIP1;1*, *CsPIP1;3*, *CsPIP2;2*, *CsPIP2;3* and *CsSIP1;1*) showed higher expression in all the seven tissues, indicating a role in constitutive transport processes throughout the plant. Others genes were found to have a low expression in all the tissues (*CsTIP1;1*, *CsTIP1;2*, *CsTIP1;3*, *CsTIP2;3*, *CsTIP5;1*, *CsTIP6;1*, *CsNIP2;1*, *CsNIP2;2*, *CsNIP2;3*, *CsNIP5;1*, *CsNIP5;2*, *CsXIP1;1* and *CsXIP2;1*). The putative tandem duplicated *CsMIP* genes were observed to have divergent expression profiles, which probably has contributed to their maintenance through regulatory subfunctionalization and neofunctionalization [49]. *CsPIP1;1* showed a higher expression than *CsPIP1;2* in all the seven tissues analyzed. *CsXIP1;1* and *CsXIP2;1* showed low expression in all the seven tissues analyzed, while *CsXIP1;2* exhibited a high expression in flower, leaf and mixed fruit tissue (Mix.3).

The cell type localization of aquaporin expression can also provide clues about their physiological roles. For instance, expression of PIP aquaporins is generally localized in organs and tissues characterized by large fluxes of water, such as vascular tissues, guard cells, flowers and fruits [4]. Their expression in roots and leaves has been also correlated with the presence of apoplastic barriers, the exodermis and endodermis in roots or in suberized bundle sheath cells in leaves, suggesting their essential role in the transmembrane water diffusion when its movement is hindered [19,20,50–55]. Except for *CsPIP2;1*, all the *CsPIPs* were found to be highly expressed in flower, leaf, fruit and mixed fruit tissues (S4 Fig), supporting their active role in the transport of water and solutes across these tissues. By contrast, the expression of TIP isoforms has been more related to developmental stages and/or organ specificity [56]. For instance, the expression of *AtTIP2;1* is especially high in the vascular system of the shoot but is barely detectable in the root [57]. *AtTIP3;1* is highly expressed in cotyledons and associated with the membrane of protein storage vacuoles [4]. TIPs are also differentially expressed during fruit maturation, e.g., the TIP1;1 homolog in pear is highly expressed in the young fruit, whereas TIP proteins levels in grape gradually increase along with ripening [58,59]. Vacuoles participate in cell expansion and, thus, their enlargement by water compartmentation is essential to provoke the rapid fruit growth that is characteristic of the ripening process. The lack of specific regulation observed along fruit ripening for PIPs isoforms points out the essential role of TIPs in this process [60]. The differential expression of *CsTIP1;3*, *CsTIP1;4*, *CsTIP2;1*, *CsTIP2;2*, *CsTIP3;1*, *CsTIP4;1* and *CsTIP5;1* highlights the functional importance of these *CsMIPs* on each tissue and stage of fruit development analyzed (S4 Fig). The overall level of *NIP* expression is usually lower than the expression of *PIPs* and *TIPs*, and it is usually associated with specialized organs and cells [10]. For instance, *AtNIP2;1* is specifically expressed in the endoplasmic reticulum (ER) of roots, whereas *AtNIP5;1* is a plasma membrane MIP mainly expressed in root elongation zones [39,61]. Our analysis showed that the *CsNIPs* had preferential expression either in flower (*CsNIP6;1*), leaf (*CsNIP2;2* and *CsNIP3;1*) or mixed fruit tissues at different developmental stages (*CsNIP1;1*, *CsNIP2;2*, *CsNIP3;1*, *CsNIP4;1* and *CsNIP6;1*) (S4 Fig). *SIPs* seem to be expressed in a range of tissues in *Arabidopsis*, including young roots, flowers and pollen [62]. It is remarkable that *SIPs* are also strongly expressed in suspension cultured cells compared to other *MIPs* [62]. *CsSIP1;1* was observed to be constitutively expressed in all the seven tissues analyzes, while the others were preferentially expressed in flower and fruit tissues (S4 Fig). *XIPs* were reported to be expressed in almost all the poplar tissues [9]. By contrast, *CsXIPs* showed a low expression in all tissues analyzed, except *CsXIP1;2*, which show a relatively high expression in flower, leaf and mixed fruit tissues at third developmental stage (Mix.3) (S4 Fig).

### Expression patterns of *CsMIP* genes under abiotic and biotic stresses

**Fig 1:**
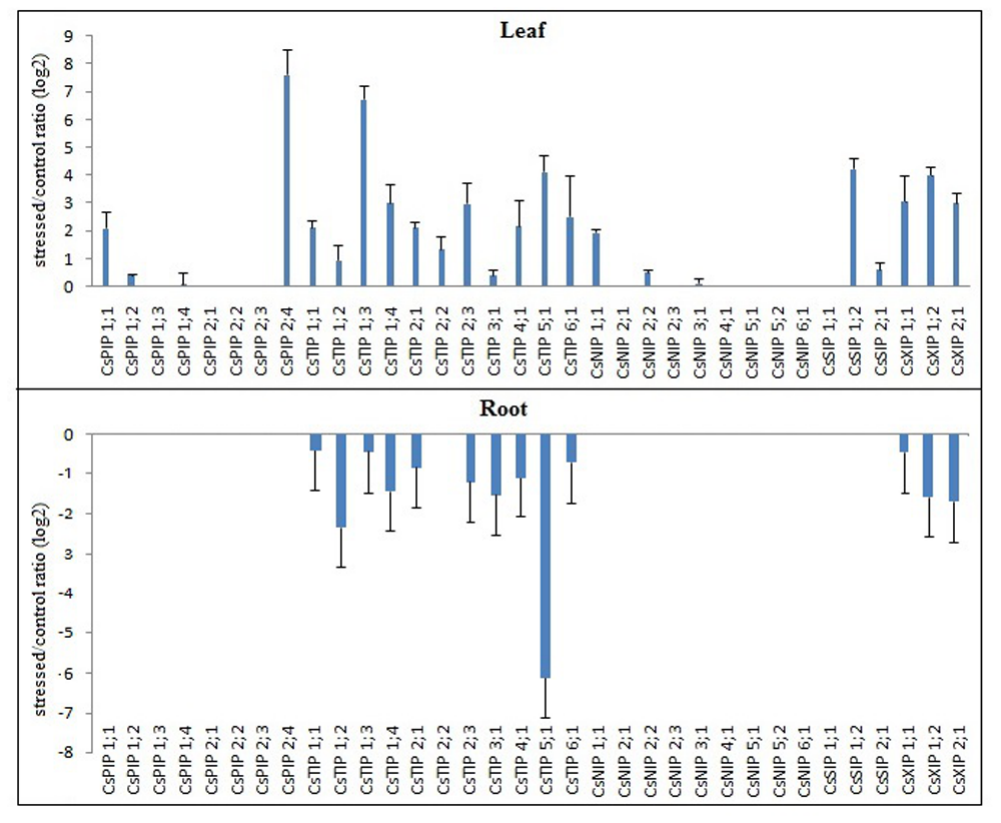
Expression analysis of the complete set of sweet orange *MIPs* in response to drought treatment. Ratios (log_2_) of relative mRNA levels between stressed and control plants for all 34 *CsMIPs* in leaves and roots, as measured by qPCR. *GAPC2* was used as an endogenous control. The bars show means ± SE from three biological replicates.

**Fig 2:**
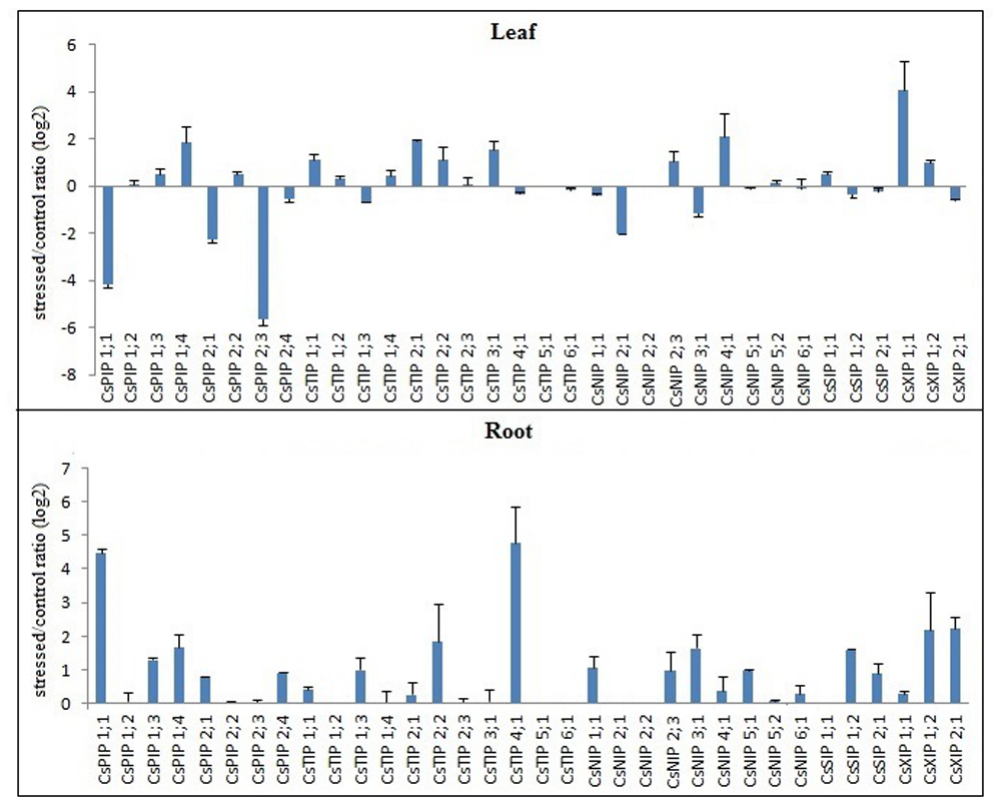
Expression analysis of the complete set of sweet orange *MIPs* in response to salt treatment. Ratios (log_2_) of relative mRNA levels between stressed and control plants for all 34 *CsMIPs* in leaves and roots, as measured by qPCR. *GAPC2* was used as an endogenous control. The bars show means ± SE from three biological replicates.

**Fig 3:**
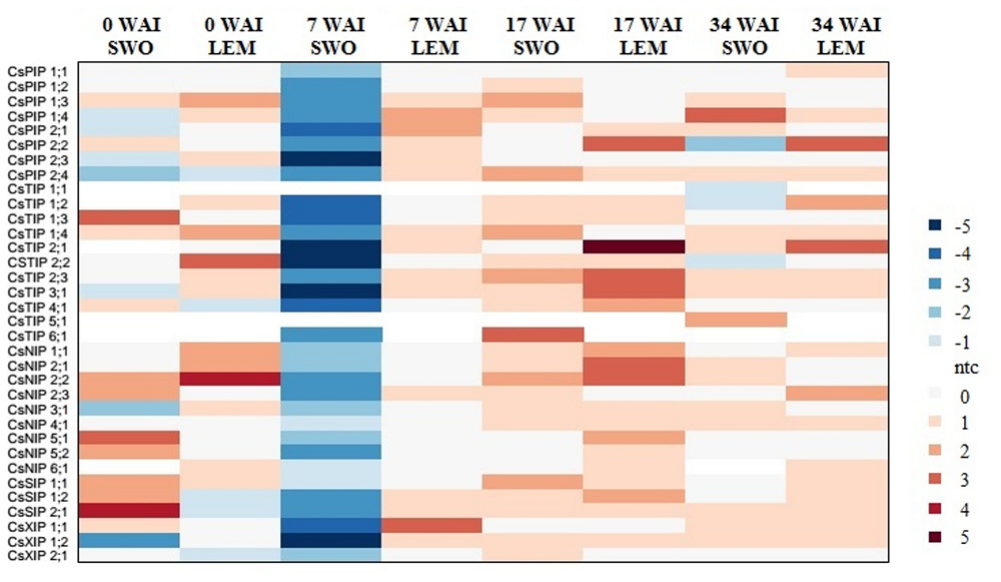
Expression analysis of the complete set of sweet orange *MIPs* in response to ‘*Ca*. L. asiaticus’ infection in rough lemon (LEM) and sweet orange (SWO). Ratios (log_2_) of relative mRNA levels between infected and control plants at 0, 7, 17, and 34 WAI for all 34 *CsMIPs*, as measured by qPCR. *GAPC2* was used as an endogenous control.

To identify *CsMIPs* with a potential role in abiotic and biotic stress response of sweet orange, the expression patterns of all the 34 sweet orange *MIPs* were investigated in plants exposed to drought, high salinity and ‘*Ca*. L. asiaticus’ (HLB) infection, by qPCR. Considering the log_2_ fold change (LFC) of ≥1.00 or ≤-1.00 as cutoff threshold between stressed and control plants, the qPCR analyses showed that all the *CsMIPs* were differentially expressed in at least one stress condition and tissue analyzed (Figs 1–3). Twelve *CsMIPs* (*CsPIP1;1*, *CsPIP2;4*, *CsTIP1;3*, *CsTIP2;1*, *CsTIP2;2*, *CsTIP3;1*, *CsTIP4;1*, *CsNIP1;1*, *CsSIP1;2*, *CsXIP1;1*, *CsXIP1;2* and *CsXIP2;1*) were observed to be differentially expressed in response to all the three stress conditions analyzed, in at least one tissue studied. Interestingly, only *CsTIP1;1* showed differential expression exclusively in response to the abiotic stress treatments, while six *CsMIPs* (*CsPIP1;2*, *CsPIP2;2*, *CsNIP2;2*, *CsNIP5;2*, *CsNIP6;1* and *CsSIP1;1*) were differentially expressed exclusively under the biotic stress treatment. These results seem to be consistent with the respective organization of the stress-responsive *cis*-acting regulatory elements observed in the *CsMIPs* promoters (S5 Fig). *CsTIP1;1* was observed to contain the highest number of ABRE copies in the promotor region among the sweet orange *MIP* genes, while no or a low number of ABRE (less than 2 copies) and other stress-responsive *cis*-acting regulatory elements was detected in the promoter regions of the *CsMIPs* that were not induced by the abiotic stress treatments (S5 Fig). A single copy of DRE/CRT has been observed to be sufficient for ABA-independent stress-responsive gene expression, while more than two ABRE sequences are usually required for the ABA-responsive transcription [63].

All the *CsTIPs* and *CsXIPs* were upregulated in leaves but downregulated in roots by drought treatment, while only two *CsPIPs* (*CsPIP1;1* and *CsPIP2;4*) and one *CsNIP* (*CsNIP1;1*) and *CsSIP* (*CsSIP1;2*) were differentially upregulated by drought treatment in leaves (Fig 1). Most *CsMIPs* were upregulated by salt treatment in roots, and either upregulated or downregulated by this treatment in leaves, depending on the isoform (Fig 2). A coordinated up- and downregulation, depending on the *MIP* gene and organ examined, has been described as a general pattern of *MIP* regulation during drought and salt stresses in *Arabidopsis* [64–67], soybean [12] and rice [68]. It has been proposed that a general downregulation of *MIPs* might be a way for the plant to minimize water loss and the hence loss of turgor in specific organs, and that the upregulation of individual *MIPs* could be a way for the plant to direct water flow to certain organs or cells that are crucial for plant survival during stress, or necessary for its fast recovery upon rehydration [65]. Thus, the concomitant upregulation in leaves and downregulation in roots of the *CsTIPs* and *CsXIPs* by drought treatment likely reveal a coordinated regulation of these *MIP* isoforms to direct the water flow through the leaf plasma membrane and tonoplast, while avoiding the water loss in roots. On the other hand, the upregulation of most *CsMIPs* in roots by salt treatment suggests their coordinated regulation to increase the overall water flow into this organ, since it is well known that salt stress reduces the hydraulic conductivity in roots, resulting in decreases of water flow from root to shoot. Those *CsMIPs* that were upregulated in leaves by salt treatment (i.e., *CsPIP1;4*, *CsTIP1;1*, *CsTIP2;1*, *CsTIP2;2*, *CsTIP3;1*, *CsNIP2;3*, *CsNIP4;1*, *CsXIP1;1* and *CsXIP1;2*) may also contribute to increase the water flow into this organ.

The time-course transcriptional analysis in response to ‘*Ca*. L. asiaticus’ infection showed that 32 out of 34 *CsMIPs* were strongly downregulated at the early stage (7 weeks) in the highly susceptible sweet orange, but not in the tolerant rough lemon, whose expression levels of all *CsMIPs* were either upregulated or unchanged at this stage (Fig 3). At the later time points (17 and 34 weeks), significantly differential expression levels between the susceptible and tolerant citrus species were essentially limited to *CsPIP2;2*, *CsTIP1;2*, *CsTIP2;1*, *CsTIP2;2* and *CsNIP5;1*. ‘*Ca*. L. asiaticus’ is the most widely distributed of the three species of the phloem-limited α-proteobacterium, designated as *Candidatus* Liberibacter (i.e., ‘*Ca*. L. asiaticus’, ‘*Ca*. L. africanus’ and ‘*Ca*. L. americanus’), which has been associated with the most destructive disease affecting citrus worldwide, Huanglongbing (HLB) [69]. Leaf symptoms of HLB disease have been well documented [69]. These include vein yellowing and blotchy mottle, reduced leaf size and premature leaf abscission. Anatomical alterations caused by the disease in leaves include the excessive accumulation of starch, callose depositions, phloem plugging, necrosis and collapse, swelling of sieve elements and companion cell walls, and the disruption of chloroplast inner grana structures [27,69–74]. Significant differences on phloem ultrastructure and phloem loading activity were also observed between HLB-infected sweet orange and rough lemon plants, highlighting some underlying differences between tolerance and susceptibility mechanisms to HLB disease [27]. While the phloem transport activity in the midribs of leaves was considerably impaired in diseased sweet orange, it was much less affected in infected rough lemon [27]. The downregulation of most *CsMIPs* as observed 7 WAI in the susceptible sweet orange, but not in the tolerant rough lemon, correlates with the onset of disease symptoms, such as blotchy leaf mottle and yellowing [27]. The continued contrasting expression patterns of *CsPIP2;2*, *CsTIP1;2*, *CsTIP2;1*, *CsTIP2;2* and *CsNIP5;1* between the tolerant and susceptible citrus species throughout the 17 and/or 34 WAI suggests an involvement of these *CsMIP* genes in further symptom development, such as growth inhibition and impaired phloem loading [27]. Taken together, these results indicate that *MIP* genes may play an active role in the pathogenesis of HLB disease by regulating the flow of water and nutrients required for the normal growth and development of citrus plants. The recent finding in a microarray experiment that *CsPIPs* were observed to be downregulated in stems of HLB-symptomatic sweet orange trees, 16 months after inoculation of ‘*Ca*. L. asiaticus’, is consistent with this interpretation [74].

## Conclusions

This study presented a genome-wide survey of the *MIP* gene family in sweet orange. A total of 34 open reading frames (ORFs) encoding MIP proteins were identified and characterized as to their sequences, phylogenetic relationships, genomic organization, tissue-specific gene expression, and expression profiles upon abiotic and biotic stresses. Our results allow us to assess the relative contribution of each *CsMIP* member to water and solute transport in different tissues and in response to drought, salinity and ‘*Ca*. L. asiaticus’ infection. These results suggest a special role for *CsTIPs* and *CsXIPs* in delivering water to the leaves while preventing root tissue dehydration under drought stress, and for most *CsMIPs* in increasing the overall water flow into the roots during salt stress and also regulating the flow of water and nutrients during the development of HLB disease. Taken together, our results support the idea that these *CsMIP* genes represent an important genetic resource for improving citrus tolerance or resistance to both abiotic and biotic stresses.

## Supporting Information

S1 Fig Phylogenetic relationships of the complete set of 34 sweet orange MIPs with members of *Arabidopsis* and other plants. The deduced amino acid sequences were aligned using ClustalW2 and the phylogenetic tree was generated using Bootstrap N-J tree (1,000 resamplings) method and MEGA program (v6.0.5). Numbers at internal nodes denotes the results of bootstrapping analysis (n = 1000). Black diamonds indicate MIP gene from sweet orange. Cs, *Citrus sinensis*; At, *Arabidopsis thaliana*; Rc, *Ricinus communis*; Nb, *Nicotiana benthamiana*.
(TIF)

S2 Fig Analysis of exon-intron structures of the 34 sweet orange *MIP* genes. NOI denotes the number of introns, E the exon and I the intron.
(TIF)

S3 Fig Chromosomal locations of *CsMIPs*. The chromosomal position of each *CsMIP* was mapped according to the *Citrus sinensis* Annotation Project (CAP). The scale is in Mb. *CsSIP* (circle), *CsPIP* (star), *CsNIP* (square), *CsTIP* (triangle), *CsXIP* (diamond).
(TIF)

S4 Fig Heatmap of the expression of *CsMIPs* in different tissues of sweet orange. Mix.1, Mix.2 and Mix.3 indicate mixed fruit tissues from different developmental stages. The heatmap was generated using R 3.1.0 software. The color scale shown represents RPKM-normalized log_2_-transformed counts.
(TIF)

S5 Fig Analysis of the stress-responsive *cis*-elements ABRE (ACGTG), DRE/CRT (G/ACCGAC), MYBS (TAACTG) and LTRE (CCGAC) in promoters of sweet orange *MIP* genes. The *cis*-elements were analyzed in the 1 kb upstream promoter region of translation start site of all *CsMIPs* using the PLACE database.
(TIF)

S1 Table Primers used in the qPCR analysis. (DOCX)

S2 Table Conserved specificity-determining amino acid residues in sweet orange MIPs. (DOCX)

S3 Table Similarity analysis of *MIP* genes from diploid (Phytozome) and dihaploid (CAP) sweet oranges (*C*. *sinensis*) and haploid Clementine (*C*. *clementina*). (DOCX)
