# Supplementary figures and images for: Genome-Wide Characterization and Expression Analysis of Major Intrinsic Proteins during Abiotic and Biotic Stresses in Sweet Orange (Citrus sinensis L. Osb.)
Source: PLoS One. 2015 Sep 23;10(9):e0138786. doi: 10.1371/journal.pone.0138786 (PMC4580632; doi:10.1371/journal.pone.0138786)

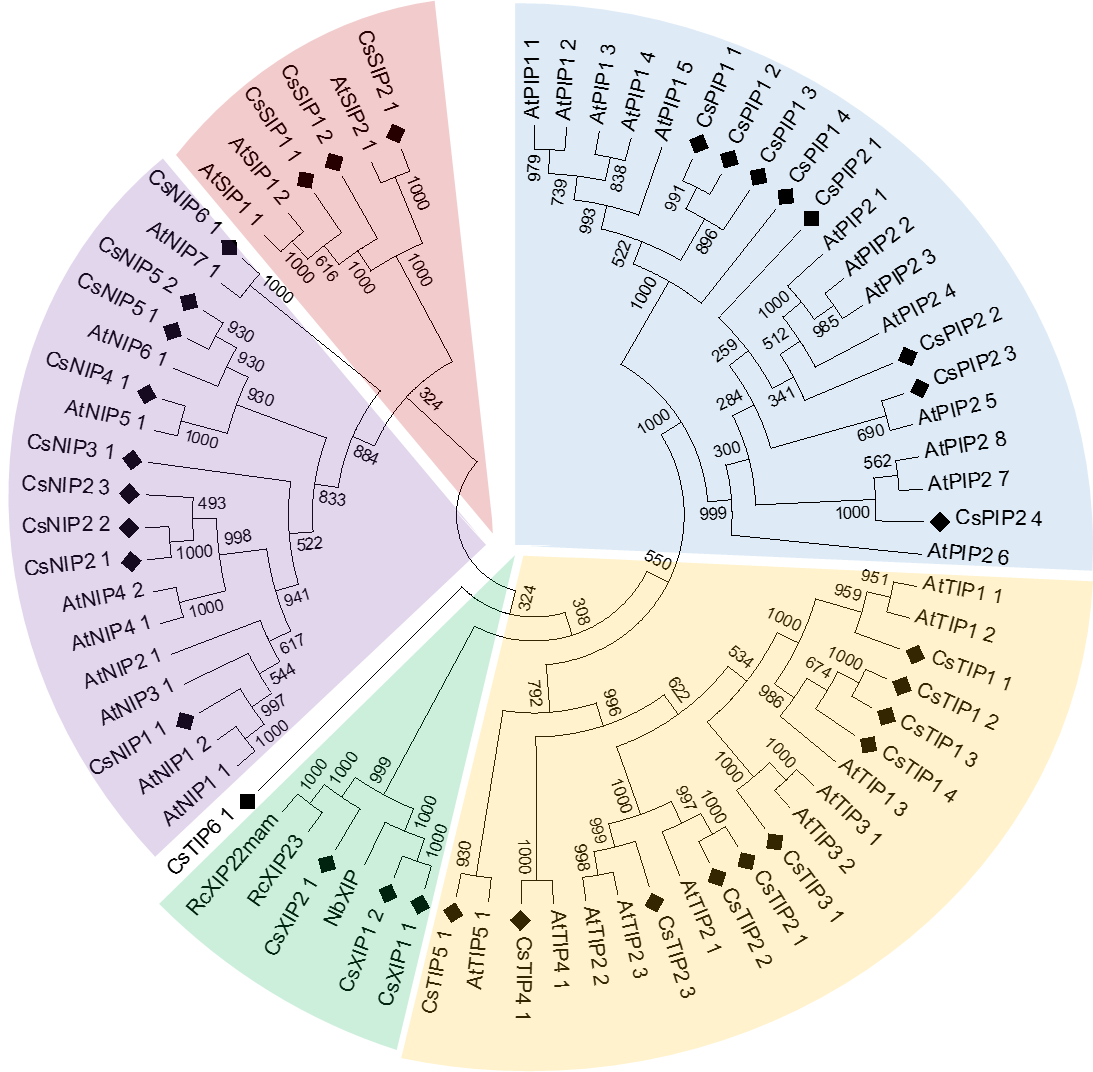

Supplement: S1 Fig — The deduced amino acid sequences were aligned using ClustalW2 and the phylogenetic tree was generated using Bootstrap N-J tree (1,000 resamplings) method and MEGA program (v6.0.5). Numbers at internal nodes denotes the results of bootstrapping analysis (n = 1000). Black diamonds indicate MIP gene from sweet orange. Cs, Citrus sinensis; At, Arabidopsis thaliana; Rc, Ricinus communis; Nb, Nicotiana benthamiana. (TIF) [file pone.0138786.s001.tif]

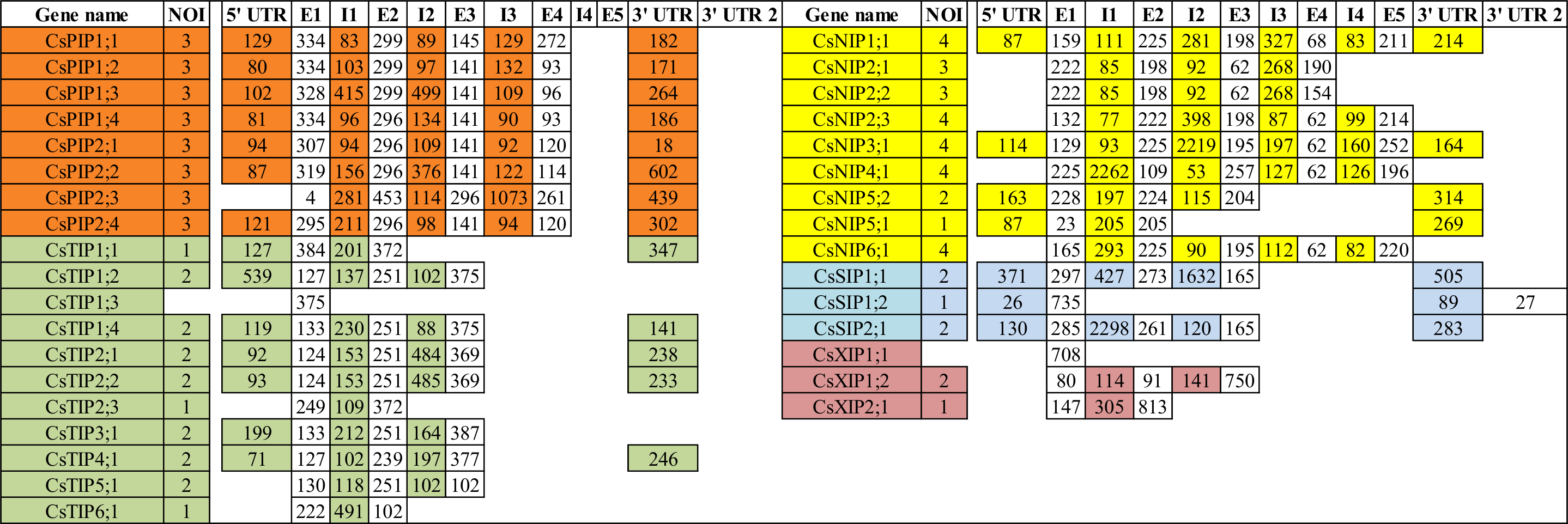

Supplement: S2 Fig — NOI denotes the number of introns, E the exon and I the intron. (TIF) [file pone.0138786.s002.tif]

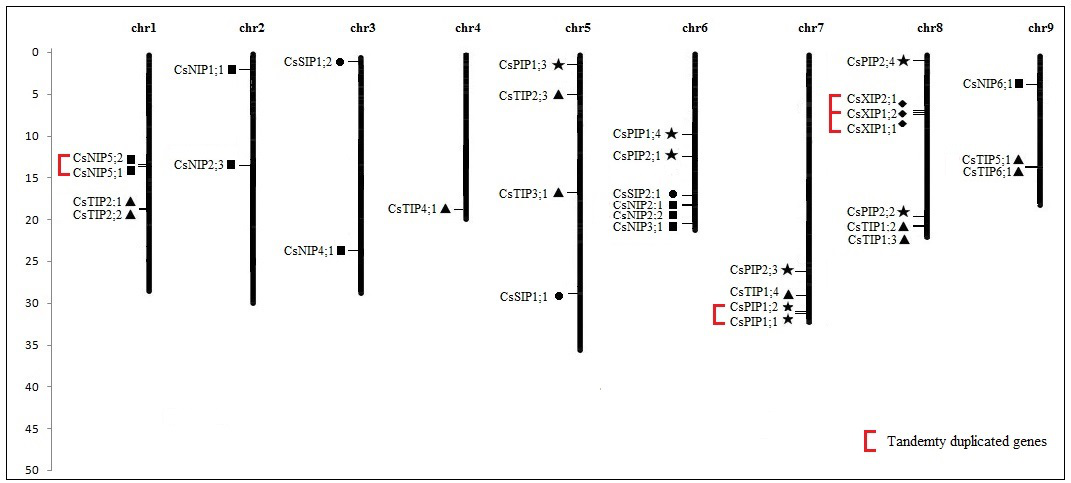

Supplement: S3 Fig — The chromosomal position of each CsMIP was mapped according to the Citrus sinensis Annotation Project (CAP). The scale is in Mb. CsSIP (circle), CsPIP (star), CsNIP (square), CsTIP (triangle), CsXIP (diamond). (TIF) [file pone.0138786.s003.tif]

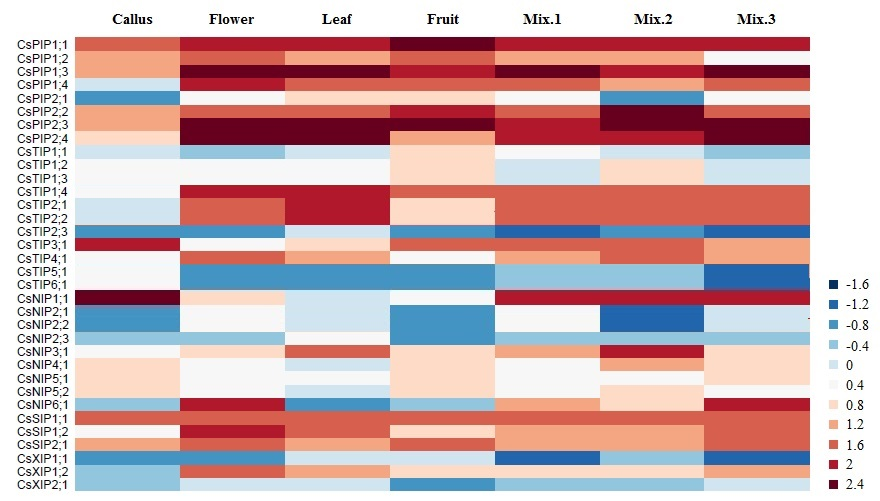

Supplement: S4 Fig — Mix.1, Mix.2 and Mix.3 indicate mixed fruit tissues from different developmental stages. The heatmap was generated using R 3.1.0 software. The color scale shown represents RPKM-normalized log2-transformed counts. (TIF) [file pone.0138786.s004.tif]

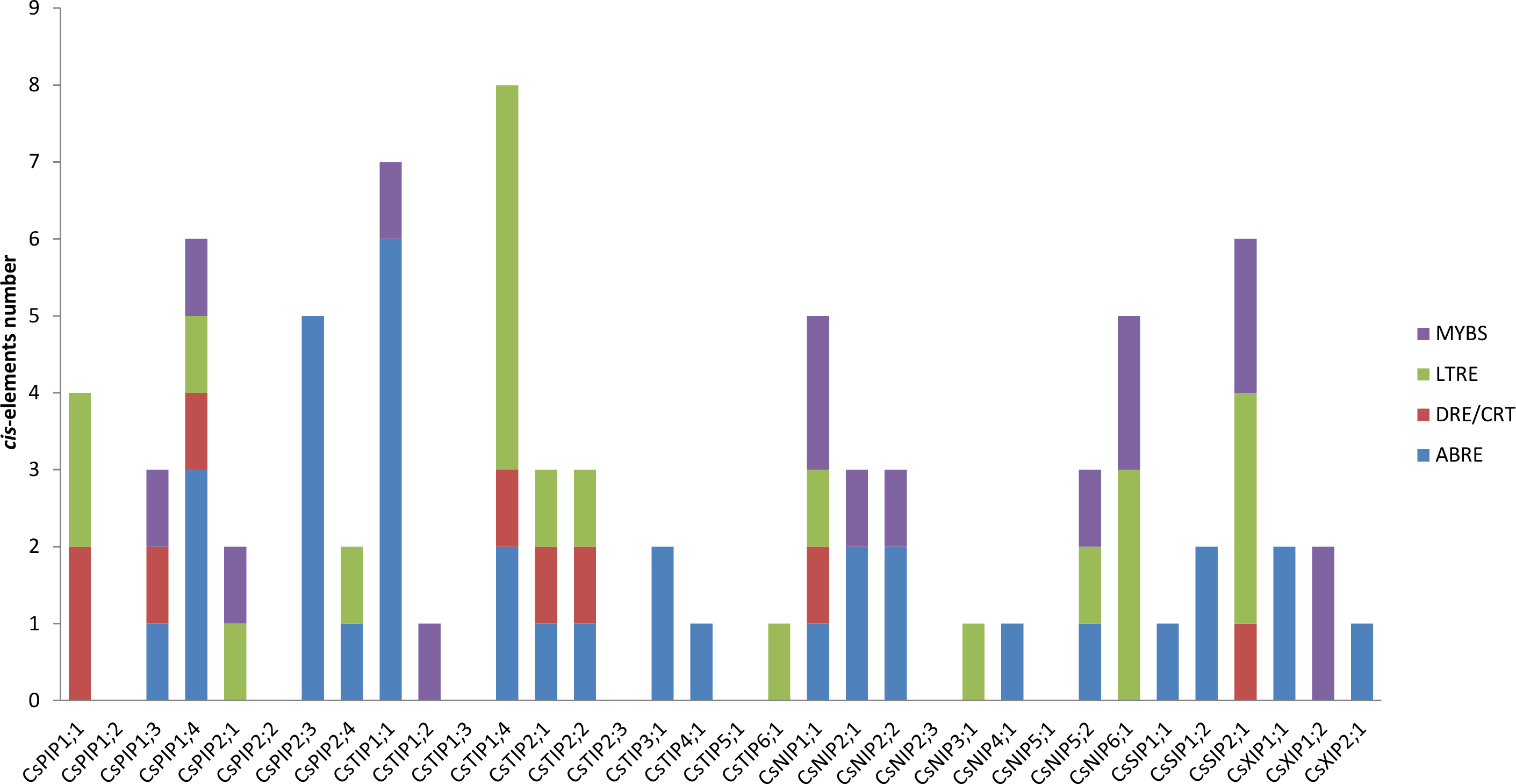

Supplement: S5 Fig — The cis-elements were analyzed in the 1 kb upstream promoter region of translation start site of all CsMIPs using the PLACE database. (TIF) [file pone.0138786.s005.tif]
