# Supplementary material for: Genome-Wide Characterization and Expression Analysis of Major Intrinsic Proteins during Abiotic and Biotic Stresses in Sweet Orange (Citrus sinensis L. Osb.)
Source: PLoS One. 2015 Sep 23;10(9):e0138786. doi: 10.1371/journal.pone.0138786 (PMC4580632; doi:10.1371/journal.pone.0138786)
Supplement: S1 Table — (DOCX) [file pone.0138786.s006.docx]

**S1 Table. Primers used in the qPCR analysis.**

| **Name** | **Locus ID** | **Primer** | **Size product (bp)** |
| --- | --- | --- | --- |
| CsPIP1;1 | orange1.1g018895 | F: 5’-CATTCTCATCACAACATCAAACG-3’  R: 5’-CTGCTAGTCCCTCAAAAACACAA-3’ | 84 |
| CsPIP1;2 | orange1.1g023021 | F: 5’-TCACTCCCGTAGCAAGATCA-3’  R: 5’-TTTCGCTCGCTCTTCTTCA-3’ | 90 |
| CsPIP1;3 | orange1.1g023107 | F: 5’-CCTCAACTTTCTCGCTACGC-3’  R: 5’-TGATGAACCTCTCTCTCGCTCT-3’ | 85 |
| CsPIP1;4 | orange1.1g023069 | F: 5’-TCTGCTGTATATGTACAACCCTTCG-3’  R: 5’-ATAGGAATCGGCCATGAACA-3’ | 80 |
| CsPIP2;1 | orange1.1g023108 | F: 5’-TAGGCGGCAATGCTAAGTTT-3’  R: 5’-ATGATGAAGAAGGGCGAAGA-3’ | 89 |
| CsPIP2;2 | orange1.1g022966 | F: 5’-GCAAACACAACAGTCGTAGCTCT-3’  R: 5’-CTTCAACATCCTTCCCCATTT-3’ | 90 |
| CsPIP2;3 | orange1.1g019681 | F: 5’-TGTTGTCATTTTGCTACTCGTTTC-3’  R: 5’-GGCGTGCCATATTGCTTTTA-3’ | 85 |
| CsPIP2;4 | orange1.1g023370 | F: 5’-TTTCTGTTATTTGTTCGCTTGTGT-3’  R: 5’-AATGGAAAAATAAAGAGAAGGGTCA-3’ | 81 |
| CsTIP1;1 | orange1.1g025548 | F: 5’-AAGCTCCTAGTTGAGAAGTGGAGA-3’  R: 5’-ACGATGAAGATTGAACCTTTGG-3’ | 81 |
| CsTIP1;2 | orange1.1g025600 | F: 5’-AACGGTCGGTTAAGCAGATT-3’  R: 5’-GAGGGCGGAGAAAATATTGA-3’ | 82 |
| CsTIP1;3 | orange1.1g037978 | F: 5’-GAGGCTCCAAAACCACAAAT-3’  R: 5’-TTTCTTAGCGCGATGGGTAT-3’ | 90 |
| CsTIP1;4 | orange1.1g025464 | F: 5’-AACGGAGCACAAAACAGAGC-3’  R: 5’-CGAAAATTTTGTCAAAGAAAAACG-3’ | 83 |
| CsTIP2;1 | orange1.1g025817 | F: 5’-TCATTTCTTTTGGAGGTTGTAAAAA-3’  R: 5’-TCCCAATCCATCCATTATCG-3’ | 80 |
| CsTIP2;2 | orange1.1g025865 | F: 5’-TGTGGTGGGGTGTATGAAAA-3’  R: 5’-TTATCTGACGAAACCCCATT-3’ | 83 |
| CsTIP2;3 | orange1.1g038895 | F: 5’-GATCTGTTTGGGCTTTTTGG-3’  R: 5’-TTAAACATGACGAGGCACAA-3’ | 83 |
| CsTIP3;1 | orange1.1g025197 | F: 5’-CGCAGCATCATCCATTAACA-3’  R: 5’-AAAGCTGCTTCTGCTTCTGC-3’ | 83 |
| CsTIP4;1 | orange1.1g025864 | F: 5’-AAGCTGCTGTTTCTCTCTTGATG-3’  R: 5’-CAAAATGACAGCAGCCAAAAA-3’ | 88 |
| CsTIP5;1 | orange1.1g046726 | F: 5’-TCTTGCGGAATTCATCTCAAC-3’  R: 5’-GCTGCGTCTGGACTCAATTT-3’ | 87 |
| CsTIP6;1 | orange1.1g042738 | F: 5’-CTTGAAATCCACGAACCTCA-3’  R: 5’-AGCCCACCAATGGAAATAAA-3’ | 85 |
| CsNIP1;1 | orange1.1g023184 | F: 5’-CCAAGAGGAGGACGCTGTT-3’  R: 5’-CCAGTACATGCCATTCACACA-3’ | 86 |
| CsNIP2;1 | orange1.1g036721 | F: 5’-TTATTGGAACGGTGACAGGA-3’  R: 5’-AGGAATTGATGTGCAGTTGG-3’ | 81 |
| CsNIP2;2 | orange1.1g040981 | F: 5’-CCATCATTCAAAAGGCCAGT-3’  R: 5’-AACCGGTTGTGCCAATAAAT-3’ | 82 |
| CsNIP2;3 | orange1.1g040755 | F: 5’-CTCCTGCCTCAACAAAATGC-3’  R: 5’-TTGCCTTTTGGAGGAGTTGA-3’ | 84 |
| CsNIP3;1 | orange1.1g023102 | F: 5’-AAGAATTCGGCTGTGTCTGT-3’  R: 5’-CACGGAATTGAAACCGTGTA-3’ | 82 |
| CsNIP4;1 | orange1.1g046511 | F: 5’-AGGCGAAGTCAGGATTCAAGT-3’  R: 5’-AACTGGAATATCCGGGAAGC-3’ | 89 |
| CsNIP5;1 | orange1.1g035030 | F: 5’-ATGAATCTGGCATTGTGCAG-3’  R: 5’-CCCGGCTATGAGTATGTTGA-3’ | 83 |
| CsNIP5;2 | orange1.1g027840 | F: 5’-CATGTGTGCGTAGCCATATGTAG-3’  R: 5’-AAATTAACGTGAAAATGCAGCAG-3’ | 87 |
| CsNIP6;1 | orange1.1g039196 | F: 5’-GTTTCTGCCTTCTGGGTTGA-3’  R: 5’-GCCAAAGCATTGAGCTTCAC-3' | 87 |
| CsSIP1;1 | orange1.1g026039 | F: 5’-AAGAAATACACGTGGCTAAAATTCA-3’  R: 5’-TTTGGTTGGGTGCCAATAAC-3’ | 82 |
| CsSIP1;2 | orange1.1g026082 | F: 5’-TCCTTCAACTCCAACAACCA-3’  R: 5’-ATGTTGCCCCAAAGTGAAAG-3’ | 84 |
| CsSIP2;1 | orange1.1g026600 | F: 5’-ATCAGCTCAGATGAAGGCAAAT-3’  R: 5’-ATTCCAAAGGGATTCATCTACAAA-3’ | 90 |
| CsXIP1;1 | orange1.1g036381 | F: 5’-ATCGACACTGGGTTTTCTGG-3’  R: 5’-TGGAGATGCTGACTTGGAAT-3’ | 88 |
| CsXIP1;2 | orange1.1g040654 | F: 5’-TTACTGTTTGTTGGGCTTGG-3’  R: 5’-TTGAAGCATACCCATCCACA-3’ | 80 |
| CsXIP2;1 | orange1.1g045670 | F: 5’-TCGCAACTATCACAGCCTTT-3’  R: 5’-GGCCTTTTTGCTAACCCTTT-3’ | 86 |
| GAPC2 | Glyceraldehyde-3-phosphate dehydrigenase C2 | F: 5’-TCTTGCCTGCTTTGAATGGA -3’  R: 5’-TGTGAGGTCAACCACTGCGACAT-3’ | 80 |
