# Supplementary material for: Genome-Wide Characterization and Expression Analysis of Major Intrinsic Proteins during Abiotic and Biotic Stresses in Sweet Orange (Citrus sinensis L. Osb.)
Source: PLoS One. 2015 Sep 23;10(9):e0138786. doi: 10.1371/journal.pone.0138786 (PMC4580632; doi:10.1371/journal.pone.0138786)
Supplement: S2 Table — (DOCX) [file pone.0138786.s007.docx]

**S2 Table. Conserved specificity-determining amino acid residues in sweet orange MIPs.**

|  | **NPA** | |  | **ar/R Filter** | | | |  | **SDP^1^** | | | | | | |
| --- | --- | --- | --- | --- | --- | --- | --- | --- | --- | --- | --- | --- | --- | --- | --- |
| **Subfamily** | **1^st^** | **2^nd^** |  | **H2** | **H5** | **LE1** | **LE2** |  | **P1** | **P2** | **P3** | **P4** | **P5** | **P6** | **P7** |
| CsPIP | NPA | NPA |  | F | H | T | R |  | Q/E/M | S | A | F/D | W/F | C/S | G |
| CsTIP | NPA | NPA |  | H/N | I | A/G | R/V |  | T/A/V | S/A | A | Y | W | V/A/M | A |
| CsNIP | NPA/S/G | NPA/V |  | W/A/G/T | V/I/S | A/G | R |  | F/L/V | S/T | A | Y/F | I/L | L/C/S | A/G |
| CsSIP | NPT/S/L | NPA |  | V/A/S | I/V/H | P/G | N/S |  | M/F | A/V | A | Y | W | G/A/T | G |
| CsXIP | N/SPL/V | NPA |  | V | V | A/V | R |  | V/M | C | A | F | W | F/V | G |

^1^Specificity determining positions according to Froger et al. [34] and Zhang et al [12].
