# Supplementary material for: Genome-Wide Characterization and Expression Analysis of Major Intrinsic Proteins during Abiotic and Biotic Stresses in Sweet Orange (Citrus sinensis L. Osb.)
Source: PLoS One. 2015 Sep 23;10(9):e0138786. doi: 10.1371/journal.pone.0138786 (PMC4580632; doi:10.1371/journal.pone.0138786)
Supplement: S3 Table — (DOCX) [file pone.0138786.s008.docx]

**S3 Table. Similarity analysis of *MIP* genes from diploid (Phytozome) and dihaploid (CAP) sweet oranges (*C. sinensis*) and haploid Clementine (*C. clementina*).**

| **Diploid Sweet Orange** | | **Dihaploid Sweet Orange** | | | | **Haploid Clementine** | | | |
| --- | --- | --- | --- | --- | --- | --- | --- | --- | --- |
| **Gene name** | **ID**  **(Phytozome)** | **ID**  **(CAP)** | **Identity(%)** | **E-value** | **Score** | **ID**  **(Phytozome)** | **Identity(%)** | **E-value** | **Score** |
| *CsPIP1;1* | orange1.1g018895 | Cs7g31420 | 98 | 0 | 2858 | Ciclev10032308m | 98 | 0 | 2874 |
| *CsPIP1;2* | orange1.1g023021 | Cs7g31410 | 100 | 0 | 2607 | Ciclev10032308m  Ciclev10032298m | 99  99 | 0  0 | 2607  2621 |
| *CsPIP1;3* | orange1.1g023107 | Cs5g03460 | 100 | 0 | 3947 | Ciclev10021502m | 98 | 0 | 3864 |
| *CsPIP1;4* | orange1.1g023069 | Cs6g07970 | 100 | 0 | 2587 | Ciclev10012384m | 99 | 0 | 2565 |
| *CsPIP2;1* | orange1.1g023108 | Cs6g11660  Cs6g11690  Cs6g11700 | 97  96  94 | 0  0  0 | 1915  1905  1832 | Ciclev10012375m  Ciclev10012379m  Ciclev10012633m | 99  92  92 | 0  0  0 | 2223  1880  1847 |
| *CsPIP2;2* | orange1.1g022966 | Cs8g16640 | 100 | 0 | 3713 | Ciclev10028975m | 99 | 0 | 3916 |
| *CsPIP2;3* | orange1.1g019681 | Cs7g25610 | 98 | 0 | 4703 | Ciclev10032302m | 98 | 0 | 5012 |
| *CsPIP2;4* | orange1.1g023370 | Cs8g02530 | 99 | 0 | 2968 | Ciclev10029003m | 99 | 0 | 3000 |
| *-* | No Homology | No Homology | - | - | - | Ciclev10003297m | - | - | - |
| *CsTIP1;1* | orange1.1g025548 | orange1.1t03005 | 100 | 0 | 2558 | Ciclev10012553m | 99 | 0 | 2558 |
| *CsTIP1;2* | orange1.1g025600 | Cs8g17900 | 99 | 0 | 1976 | Ciclev10029134m | 99 | 0 | 2713 |
| *CsTIP1;3* | orange1.1g037978 | Cs8g17900 | 97 | 0 | 1062 | Ciclev10029134m | 97 | 4e-180 | 632 |
| *CsTIP1;4* | orange1.1g025464 | Cs7g28650 | 99 | 0 | 2351 | Ciclev10032534m | 99 | 0 | 2365 |
| *CsTIP2;1* | orange1.1g025817 | Cs1g15440 | 97 | 0 | 2888 | Ciclev10026351m | 100 | 0 | 3086 |
| *CsTIP2;2* | orange1.1g025865 | Cs1g15440 | 100 | 0 | 3081 | Ciclev10026351m | 97 | 0 | 2879 |
| *CsTIP2;3* | orange1.1g038895 | Cs5g08710 | 99 | 0 | 1293 | Ciclev10021867m | 99 | 0 | 1290 |
| *CsTIP3;1* | orange1.1g025197 | Cs5g17210 | 100 | 0 | 2403 | Ciclev10021799m | 97 | 0 | 2262 |
| *CsTIP4;1* | orange1.1g025864 | Cs4g19580 | 99 | 0 | 2378 | Ciclev10009294m | 99 | 0 | 2392 |
| *CsTIP5;1* | orange1.1g046726 | Cs9g14770 | 99 | 0 | 1237 | Ciclev10006865m | 99 | 0 | 1263 |
| *CsTIP6;1* | orange1.1g042738 | Cs9g14770 | 100 | 5E-114 | 412 | Ciclev10006865m | 97 | 0 | 1346 |
| *-* | No Homology | No Homology | - | - | - | Ciclev10023306m | - | - | - |
| *CsNIP1;1* | orange1.1g023184 | Cs2g04370 | 100 | 0 | 3500 | Ciclev10016171m | 96 | 0 | 3231 |
| *CsNIP2;1* | orange1.1g036721 | Cs6g17690 | 100 | 0 | 2016 | Ciclev10013768m | 97 | 0 | 1887 |
| *CsNIP2;2* | orange1.1g040981 | Cs6g17690 | 98 | 0 | 1852 | Ciclev10013768m | 99 | 0 | 1945 |
| *CsNIP2;3* | orange1.1g040755 | Cs2g16610 | 98 | 0 | 2580 | Ciclev10017700m | 98 | 0 | 2580 |
| *CsNIP3;1* | orange1.1g023102 | Cs6g21290 | 100 | 0 | 2017 | Ciclev10012382m | 97 | 0 | 5222 |
| *CsNIP4;1* | orange1.1g046511 | Cs3g20790 | 100 | 2.00E-156 | 1423 | Ciclev10001994m | 99 | 0 | 3113 |
| *CsNIP5;1* | orange1.1g035030 | Cs1g11150 | 98 | 0 | 1136 | Ciclev10026151m | 99 | 0 | 1409 |
| *CsNIP5;2* | orange1.1g027840 | Cs1g11140 | 97 | 0 | 2396 | Ciclev10026151m | 99 | 0 | 2533 |
| *CsNIP6;1* | orange1.1g039196 | Cs9g06260 | 100 | 0 | 2598 | Ciclev10005554m | 97 | 0 | 2495 |
| *-* | No Homology | No Homology | - |  |  | Ciclev10003256m | - | - | - |
| *CsSIP1;1* | orange1.1g026039 | Cs5g26100 | 99 | 0 | 6557 | Ciclev10021916m | 99 | 0 | 6482 |
| *CsSIP1;2* | orange1.1g026082 | Cs3g01900 | 100 | 0 | 1579 | Ciclev10005734m | 99 | 0 | 1553 |
| *CsSIP2;1* | orange1.1g026600 | Cs6g16190 | 100 | 0 | 1789 | Ciclev10012628m | 98 | 0 | 4933 |
| *CsXIP1;1* | orange1.1g036381 | Cs8g08830 | 99 | 0 | 1170 | Ciclev10029916m | 99 | 0 | 1272 |
| *CsXIP1;2* | orange1.1g040654 | Cs8g08820 | 100 | 0 | 2099 | Ciclev10028106m | 99 | 0 | 2049 |
| *CsXIP2;1* | orange1.1g045670 | Cs8g08810 | 100 | 0 | 2283 | Ciclev10028106m | 99 | 0 | 2208 |
